# A Video Decision Aid for the West Virginia POLST: A Randomized, Controlled Trial

**DOI:** 10.1093/geroni/igaa057.1342

**Published:** 2020-12-16

**Authors:** Cherish Heard, Emma Pino, Jarred V Gallegos, Barry Edelstein, Alvin Moss

**Affiliations:** 1 West Virginia University, Morgantown, West Virginia, United States; 2 WVU, Morgantown, United States; 3 WVU, Morgantown, West Virginia, United States

## Abstract

Patients with serious medical conditions are faced with making decisions about treatments related to end-of-life care. The Physician Orders for Life Sustaining Treatment (POLST) is a document that allows patients to express preferences for four medical decisions including cardiopulmonary resuscitation, level of medical intervention, and medically administered fluids and nutrition. The purpose of the study was to develop and evaluate a video decision aid for the West Virginia POLST form. Sixty-four community-dwelling adults (50+) were recruited to participate. Participants were randomized to active control (exercise video) or intervention group (POLST video). Knowledge and decisional conflict were measured pre- and post-intervention. Participants were given a hypothetical vignette with medical information for the purpose of completing study measures. Separate MANCOVAs were conducted to explore the relation between treatment group and decisional outcomes at post-intervention for each medical decision, while controlling for numeracy and pre-intervention ratings of knowledge and decisional conflict. Results identified significant main effects of treatment group for each of the four medical decisions. Participants who viewed the video were more knowledgeable regarding CPR F(1,59) = 42.844, p<.001, medical interventions F(1,59) = 20.475, p<.001, and medically administered fluids F(1,59) = 31.004, p<.001, compared to participants assigned to the control group. Additionally, participants who viewed the video had less decisional conflict related to CPR F(1,59) = 17.892, p<.001, medical interventions F(1,59) = 31.017, p<.001, medically administered fluids F(3,56) = 11.718, p=.001, and nutrition F(1,59) = 16.411, p<.001, compared to participants in the control group. Conclusions and practical implications will be discussed.

